# Temporal Muscle Thickness as a New Biomarker: Its Association With Alzheimer Pathology and Cognitive Impairment in Chinese Adults

**DOI:** 10.1002/jcsm.70030

**Published:** 2025-07-28

**Authors:** Yunxia Zhu, Yingying Ke, Chenxi Ren, Jiehua Zhu, Zhen Zhang, Liang Cui, Yuehua Li, Fang Xie, Qihao Guo

**Affiliations:** ^1^ Department of Gerontology Shanghai Sixth People's Hospital Affiliated to Shanghai Jiao Tong University School of Medicine Shanghai China; ^2^ Department of Radiology Shanghai Sixth People's Hospital Affiliated to Shanghai Jiao Tong University School of Medicine Shanghai China; ^3^ PET Center, Huashan Hospital Fudan University Shanghai China

**Keywords:** Alzheimer's disease, cognitive function, PET, tau pathology, temporal muscle thickness

## Abstract

**Background:**

The association between skeletal muscle mass loss and cognitive decline remains unclear. Temporal muscle thickness (TMT) has been validated as an easily obtainable surrogate marker for muscle mass loss in neurological patients. This study was aimed at exploring whether a decline in TMT correlates with Alzheimer's disease (ad)–related cognitive impairment and its underlying mechanisms.

**Methods:**

This study was a cross‐sectional substudy of the Chinese preclinical ad study, which included data from 801 participants. TMT was measured using cranial magnetic resonance imaging. Appendicular skeletal muscle index (ASMI) was estimated using an anthropometric equation as a control. Cognitive function was assessed using a battery of standardised neuropsychological tests, and participants were categorised into three groups based on clinical diagnosis: normal cognition (NC), mild cognitive impairment (MCI) and ad. Functional performance was evaluated using the Functional Activities Questionnaire. Of the 801 participants, 733 underwent ^18^F‐florbetapir PET scans to assess cerebral amyloid‐beta (Aβ) deposition. Additionally, 165 Aβ‐PET‐positive participants were randomly selected for further evaluation of cerebral tau burden using ^18^F‐MK6240 PET.

**Results:**

Significant differences in TMT measurements were observed by age and sex, with thinner TMT found in females and older adults. TMT was significantly associated with ASMI, handgrip strength and mobility. Regarding cognition, TMT, but not ASMI, was thinner in MCI and AD participants than those with NC. TMT was positively correlated with Montreal Cognitive Assessment‐Basic (MoCA‐B) scores (*r* = 0.124, *p* = 0.001), independent of confounders such as age, sex, education level, nutritional risk, history of hypertension and apolipoprotein E ε4 genotype. Among cognitive domains, TMT was most closely associated with performance on the Auditory Verbal Learning Test recognition (*p* < 0.05). Stratified analyses revealed that the correlation between TMT and MoCA‐B remained significant in MCI participants but not in AD participants. Multivariable logistic regression analysis showed that higher TMT was significantly associated with a lower risk of MCI (OR: 0.494, 95% CI: 0.287–0.849, *p* < 0.05) and a reduced likelihood of functional dependence (OR: 0.791, 95% CI: 0.670–0.935, *p* < 0.01). Furthermore, TMT was lower in participants with higher brain tau deposition and was negatively associated with tau PET Braak stages (both *p* < 0.01).

**Conclusions:**

A decline in TMT was associated with worse cognitive function, increased cerebral tau deposition and impaired functional performance. TMT may serve as a simple, noninvasive and cost‐effective marker for MCI due to AD.

## 1 Introduction

Sarcopenia is a progressive and generalised skeletal muscle disorder characterised by the age‐related loss of muscle mass, strength and function, resulting in a reduced quality of life and an increased risk of mortality in older adults [1]. It is particularly prevalent among individuals with cognitive impairment, such as those with mild cognitive impairment (MCI) and Alzheimer's disease (ad) [2]. Emerging evidence from cross‐sectional and longitudinal studies suggests that sarcopenia may be a risk factor for cognitive dysfunction, potentially serving as a prodromal state for neurodegenerative diseases [2]. However, the exact relationship between muscle mass loss and cognitive performance remains poorly understood.

Despite its clinical relevance, the evaluation of muscle mass has not yet been integrated into routine clinical workflows, as it often requires additional examinations and increases healthcare costs. Therefore, a simple, quantitative and objective tool to assess skeletal musculature status is urgently needed. Notably, temporal muscle thickness (TMT) has emerged as such an innovative surrogate indicator in view of a reliable correlation with sarcopenia‐related traits such as handgrip strength (HGS), skeletal muscle mass and gait speed [3, 4]. Importantly, TMT is easily accessible in patients with cognitive impairment since cranial magnetic resonance imaging (cMRI) is routinely performed for diagnostic purposes. However, only a few studies have preliminarily investigated the association between TMT and cognitive performance, yielding inconsistent results [3, 5, 6].

The potential mechanisms underlying the link between muscle mass loss and cognitive impairment in older adults remain poorly understood. In addition to well‐established factors such as aging, reduced physical activity, oxidative stress, neuromuscular injury, hormone dysregulation, insulin resistance and chronic inflammation [7, 8, 9], recent studies suggest that early neurodegenerative changes in the brain may also play a role [10]. For instance, Kim et al. demonstrated that lower skeletal muscle mass in nondemented older adults was associated with brain atrophy, potentially mediated by amyloid‐beta (Aβ) retention, ultimately resulting in cognitive decline [10]. The hallmark pathology of ad includes the accumulation of Aβ plaques and neurofibrillary tangles, caused by the excessive phosphorylation of tau protein, resulting in progressive neuronal death, cognitive deterioration and impaired daily functioning [11]. Neuroimaging biomarkers, particularly tau positron emission tomography (PET), have demonstrated predictive value for cognitive decline across the ad spectrum, from cognitively unimpaired individuals to those with AD [12, 13].

In this study, we used data from the Chinese preclinical Alzheimer's disease study (C‐PAS) to investigate the association between MRI‐based TMT and cognition in middle‐aged and older adults. Our objectives were (i) to evaluate the correlation between TMT and sarcopenia‐defining parameters; (ii) to assess the relationship between TMT and both cognitive and functional performance, with cognition measured using standardised neuropsychological tests and functional performance assessed using the Functional Activities Questionnaire (FAQ) and (iii) to explore the potential underlying mechanisms, focusing on the amyloid‐tau pathology of AD. We hypothesised that TMT could serve as a convenient screening tool for sarcopenia and an early indicator of cognitive decline and functional dependence. Additionally, the specific association between sarcopenia, as assessed by TMT, and AD pathology may provide insights into the muscle–brain interactions in AD.

## 2 Methods

### 2.1 Study Participants

This study included 2685 participants from the C‐PAS, with enrolment occurring between 1 December 2018 and 31 October 2022. Detailed information on the C‐PAS cohort, including inclusion/exclusion criteria, sample collection protocols and clinical marker extraction (demographics, apolipoprotein E genotype [APOE], neuropsychological test scores, diagnostic and grouping criteria and PET imaging markers), is provided in a prior publication [14]. Of the screened participants, 801 met the eligibility criteria for analysis. Exclusion criteria included cognitive impairment due to significant neurological diseases (e.g., stroke, subdural hematomas, brain trauma, epilepsy, hydrocephalus, intracranial infections and tumours), other conditions affecting cognitive function (e.g., drug abuse, alcohol dependency, abnormalities in folic acid, vitamin B12 or thyroid function) and failure to complete cMRI. Of these eligible participants, 733 underwent the detection of brain Aβ, and 165 Aβ‐PET‐positive individuals were randomly selected for further evaluation of tau burden using tau PET imaging. The study design along with participants enrolment process is depicted in Figure S1. The study was approved by the Ethics Committee of Shanghai Jiao Tong University Affiliated Sixth People's Hospital (Approval Number 2019‐041) and conducted in accordance with the Declaration of Helsinki. Written informed consent was obtained from all participants.

### 2.2 Clinical Data and Cognitive Assessment

Collected clinical data included age, sex, height, weight, years of education, smoking status, alcohol consumption and a history of chronic diseases. Body mass index (BMI) was calculated as weight (kilograms) divided by height squared (square metres). Physical activity was assessed through self‐reported weekly exercise frequency. Nutritional risk was evaluated using the Mini‐Nutritional Assessment Short‐Form (MNA‐SF). The APOE ε4 genotype was determined, and participants were classified as either APOE ε4 carriers or noncarriers. General cognitive performance was assessed using the Chinese version of the Montreal Cognitive Assessment‐Basic (MoCA‐B) and Addenbrooke's Cognitive Examination (ACE‐III). Domain‐specific cognitive tests, including the Auditory Verbal Learning Test (AVLT) 30‐min delayed free recall and recognition, the Animal Verbal Fluency Test (AFT) and the Boston Naming Test (BNT), were carried out as described in prior studies [15, 16]. HGS was measured thrice per hand using a dynamometer (EH101; Camry, China), with the maximum value recorded. Functional mobility was assessed using the Timed ‘Up and Go’ (TUG) Test [17]. Cognitive diagnoses were categorized as normal cognition (NC), MCI or ad using the National Institute on Aging‐Alzheimer's Association (NIA‐AA) 2011 criteria for ad [18] and Bondi et al.'s criteria for MCI [19]. Individuals who did not meet the criteria for MCI or ad were classified as NC. Functional performance was assessed using the FAQ, with scores ≥ 5 indicating dependence [20].

### 2.3 TMT Measurement and Appendicular Skeletal Muscle Index (ASMI) Estimation

For TMT assessment, T1‐weighted MRI scans were used. A well‐trained researcher, blinded to clinical participant characteristics, retrospectively measured TMT using Mango Version 4.1 (available at http://ric.uthscsa.edu/mango). To ensure intrarater reliability, 50 random images were remeasured. Briefly, the magnetic resonance plane was aligned parallel to the anterior commissure–posterior commissure line. Measurements were conducted at the level of the orbital roof (craniocaudal landmark and the lateral sulcus (anterior–posterior landmark), perpendicular to the long axis of the temporal muscle [21]. Images underwent quality checks, and those with motion artefacts or poor quality were excluded. Preprocessing steps included movement correction and intensity normalisation. TMT measurements were performed on both sides, and the mean value was calculated to account for potential influences of oral and dental diseases [22]. For the ASMI, appendicular skeletal muscle mass (ASM) was estimated using a previously validated equation for the Chinese population [23]: ASM = 0.193 × body weight (kg) + 0.107 × height (cm) − 4.157 × sex (1 for male, 2 for female) − 0.037 × age (yr) − 2.631. Subsequently, ASMI was calculated using ASM divided by the square of height in metres.

### 2.4 Amyloid and Tau PET Imaging

Brain Aβ and tau burden were assessed using ^18^F‐florbetapir and ^18^F‐MK6240 PET scans, performed on a PET/CT system (Biograph mCT Flow PET/CT, Siemens, Germany). Cerebral amyloid PET scans were conducted 50 min after the intravenous injection of 7.4 MBq/kg (0.2 mCi/kg) florbetapir and lasted for 20 min. Positive ^18^F‐florbetapir PET images were defined via visual rating according to established guidelines for amyloid PET interpretation [24]. For tau PET imaging, 5.55 MBq/kg ^18^F‐MK6240 was injected intravenously in a bolus, and a 20‐min acquisition began 90 min postinjection. Images were reconstructed via filtered back projection and preprocessed using statistical parametric mapping (SPM) 12 software. The inferior cerebellum served as the reference region for calculating the standard uptake value ratio (SUVR). Tau positivity was defined as 2.5 standard deviations above the mean SUVR of young, cognitively unimpaired individuals for each region of interest (ROI) [25]. PET Braak‐like stages were classified as follows: Braak I (transentorhinal), Braak II (entorhinal and hippocampus), Braak III (amygdala, parahippocampal gyrus, fusiform gyrus and lingual gyrus), Braak IV (insula, inferior temporal, lateral temporal, posterior cingulate and inferior parietal), Braak V (orbitofrontal, superior temporal, inferior frontal, cuneus, anterior cingulate, supramarginal gyrus, lateral occipital, precuneus, superior parietal, superior frontal and rostromedial frontal) and Braak VI (paracentral, postcentral, precentral and pericalcarine) [26]. The cut‐offs for each stage were established as follows: 1.05 (Braak I), 1.11 (Braak II), 1.17 (Braak III), 1.23 (Braak IV), 1.21 (Braak V) and 1.35 (Braak VI). Braak‐like stages were identified only if the current and preceding stages were met, while later stages were considered negative [25]. The interpretation of PET images was independently performed by three nuclear medicine specialists, with results depending on the consensus of at least two of them.

### 2.5 Identification of Confounders

To identify potential confounders affecting the relationship between TMT and cognitive function, a linear regression model was built using the MoCA‐B score as the dependent variable and TMT as the independent variable. Variables of interest were sequentially added as covariates, and the coefficient of TMT in the adjusted model was compared to that in the original model. Bootstrap resampling, performed with 1000 iterations, was used to calculate the confidence interval (CI) for the coefficient difference. A CI that did not cross zero indicated a significant confounding effect.

### 2.6 Statistical Analysis

Continuous variables were assessed for normality using the Shapiro–Wilk test and presented as means ± standard deviations or medians with interquartile ranges, depending on their distribution. Between‐group differences were analysed using two‐tailed Student's *t*‐tests, Kruskal–Wallis *H* tests or Mann–Whitney *U* tests, as appropriate. Categorical variables, expressed as frequencies and percentages, were analysed with chi‐square tests. Spearman's correlation was used to assess the association between TMT and age, as well as sarcopenia‐defining parameters. Partial correlation analyses, adjusted for age, sex and education, were conducted to examine the relationships between TMT, ASMI and neuropsychological test scores. A further partial correlation model, adjusted for additional covariates (MNA‐SF score, history of hypertension and APOE genotype), was performed to explore the correlation of TMI and ASMI with MoCA‐B in the total sample and subgroups stratified by age, sex, diagnosis (NC, MCI and ad), APOE genotype and brain Aβ burden. Associations between TMT quartiles and cognitive decline (MCI and ad) were assessed using multivariable binary logistic regression, with results reported as adjusted odds ratios (ORs) and 95% CIs. Potential mechanisms were explored by correlating TMT with tau PET Braak‐like stages and SUVR values using Spearman's correlation. Finally, the association between TMT and functional outcome, as assessed by the FAQ, was analysed using binary logistic regression, both in crude and adjusted models (accounting for age, sex, education, HGS and TUG test results). All statistical analyses were performed using SPSS 26.0 (SPSS Inc., Chicago, Illinois). *p* < 0.05 was considered statistically significant.

## 3 Results

### 3.1 Characteristics of Enrolled Participants

A total of 801 participants (307 males and 494 females) with an average age of 65.2 ± 7.8 years were enrolled. Among them, 394 (49.2%) were categorized as NC, 260 (32.5%) had MCI and 147 (18.4%) had AD. Notably, participants with MCI (*p* < 0.05) and AD (*p* < 0.01) were older than the NC ones. Cognitive impairment was more pronounced in participants with lower education levels and higher nutritional risk (both *p* < 0.001). AD patients also had a lower BMI compared to the NC group (*p* < 0.05). As predicted, significant differences were observed across neuropsychological test scores (e.g., MoCA‐B and ACE‐III), APOE ε4 positivity rates and AD PET imaging findings (Aβ and tau deposition) as cognitive impairment progressed (all *p* < 0.001). ASMI, an indicator of skeletal muscle mass, showed no significant differences across the groups. However, the TMT, a convenient surrogate marker for low skeletal muscle mass in neurological patients, was significantly lower in MCI (*p* < 0.05) and AD (*p* < 0.001) compared to NC. Additionally, participants with cognitive decline exhibited lower HGS (both *p* < 0.05) and longer TUG times (both *p* < 0.001) compared to NC individuals. No significant differences in sex, smoking or drinking habits, physical activity or history of chronic diseases were observed among the groups. The overall demographic and clinical details are presented in Table 1.

**TABLE 1:** Demographic and clinical characteristics of the participants according to cognitive status (*n* = 801).

Variable	NC (*n* = 394)	MCI (*n* = 260)	ad (*n* = 147)	*p*
Age (years)	64.2 (8.1)	65.7 (6.9)*	66.7 (8.3)**	**0.002**
Sex (male, *n*, %)	144 (36.5)	99 (38.1)	64 (43.5)	0.329
Education (years)	13 (10–15)	11 (9–13)**	9 (6–12)**, ^††^	**< 0.001**
Smoking (*n*, %)	51 (13.9)	37 (15.2)	26 (18.2)	0.472
Alcohol consumption (*n*, %)	71 (19.3)	40 (16.3)	33 (23.2)	0.246
BMI (kg/m^2^)	23.8 (3.1)	23.8 (3.1)	23.2 (3.1)*	0.095
Physical activity status (≥ 3×/week, *n*, %)	117 (57.9)	100 (66.2)	62 (59.6)	0.269
MNA‐SF	11 (11–11)	11 (10–11)**	11 (9–11)**, ^††^	**< 0.001**
History (*n*, %)				
Hypertension	125 (32.5)	86 (34.0)	49 (33.6)	0.917
Diabetes mellitus	57 (14.5)	35 (13.7)	26 (17.8)	0.522
Hyperlipidemia	74 (19.0)	45 (17.7)	20 (13.7)	0.360
Coronary heart disease	26 (6.6)	14 (5.5)	5 (3.4)	0.354
APOE ε4 positive (*n*, %)	77 (19.5)	73 (28.1)*	68 (46.3)**, ^††^	**< 0.001**
Neuropsychological tests				
MoCA‐B	26 (24–27)	22 (20–24)**	13 (9–16)**, ^††^	**< 0.001**
ACE‐III	83 (78–88)	71 (66–77)**	50 (40–58)**, ^††^	**< 0.001**
AVLT delayed recall	5 (4–7)	2 (1–3)**	0 (0–1)**, ^††^	**< 0.001**
AVLT recognition	22 (21–23)	19 (17–21)**	16 (13–18)**, ^††^	**< 0.001**
BNT	25 (23–27)	22 (19–25)**	19 (15–22)**, ^††^	**< 0.001**
AFT	17 (15–20)	13 (11–15)**	10 (7–12)**, ^††^	**< 0.001**
ad PET imaging				
Aβ PET (+) (*n*, %)	107 (28.2)	101 (39.8)*	109 (77.9)**, ^††^	**< 0.001**
Tau PET (+) (*n*, %)	4 (6.1)	19 (35.8)**	34 (73.9)**, ^††^	**< 0.001**
Sarcopenia components				
Mean TMT (mm)	6.03 (5.02–7.16)	5.76 (4.58–6.61)*	5.32 (4.33–6.41)**	**< 0.001**
ASMI (kg/m^2^)	6.44 (5.77–7.61)	6.42 (5.84–7.51)	6.77 (5.82–7.54)	0.985
HGS (kg)	24.3 (19.9–31.7)	22. 7 (18.7–28.8)*	20.1 (16.3–26.7)**	**< 0.001**
TUG (s)	9.0 (8.0–10.0)	10.0 (8.6–11.5)**	12.0 (9.7–13.8)**, ^††^	**< 0.001**

*Note:* Statistically significant comparisons are bolded. Normally distributed continuous values are presented as mean ± standard deviation, nonnormally distributed continuous variables are given as median (25% and 75% interquartile) and categorical variables are given as frequency (percentage). *p* values were calculated by ANOVA for normally distributed continuous variables, the Kruskal–Wallis test for skewed distributed continuous variables and by chi‐square test for categorical variables.

Abbreviations: Aβ, amyloid‐β; ACE‐III, Addenbrooke's Cognitive Examination; ad, Alzheimer's disease; AFT, Animal Verbal Fluency Test; APOE, apolipoprotein E; ASMI, appendicular skeletal muscle index; AVLT, Auditory Verbal Learning Test; BMI, body mass index; BNT, Boston Naming Test; HGS, handgrip strength; MCI, mild cognitive impairment; MNA‐SF, Mini‐Nutritional Assessment Short‐Form; MoCA‐B, Montreal Cognitive Assessment‐Basic; NC, normal cognition; PET, positron emission tomography; TMT, temporal muscle thickness; TUG, Timed ‘Up and Go’ Test.

* *p* < 0.05 using post hoc analyses, comparing MCI and NC groups or ad and NC groups.

** *p* < 0.01 using post hoc analyses, comparing MCI and NC groups or ad and NC groups.

†† *p* < 0.01 using post hoc analyses, comparing MCI and ad groups.

### 3.2 TMT Was Associated With Age, Sex and Sarcopenia‐Defining Parameters

**FIGURE 1:**
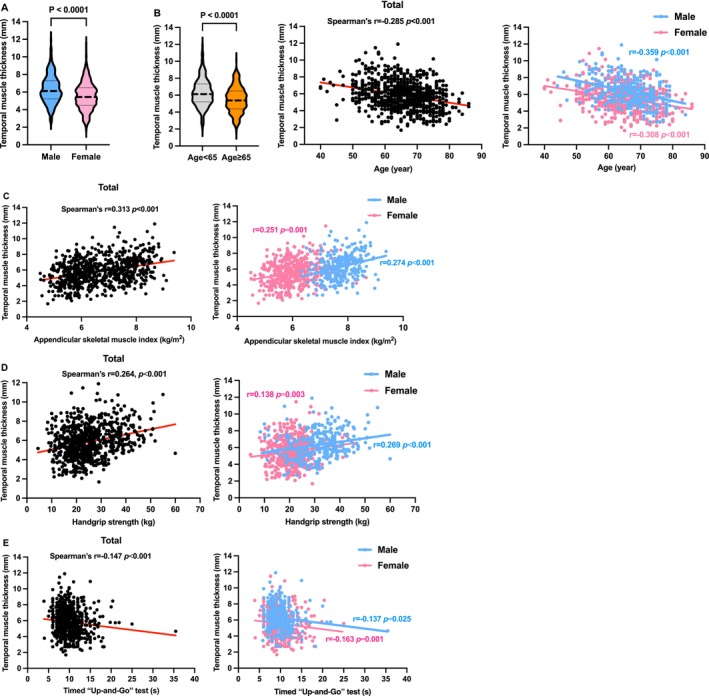
Association between temporal muscle thickness and sex (A), age (B) and sarcopenia‐defining parameters (C–E).

We observed inherent sex‐ and age‐related differences in TMT (Figure 1A,B), with males and younger participants having thicker TMT compared to females and older participants. Spearman's correlation analysis revealed an inverse relationship between TMT and age in both sexes (male: *r* = −0.359, female: *r* = −0.308; both *p* < 0.001). Further examination of TMT's relationship with sarcopenia‐related parameters showed significant correlations (Figure 1C–E): TMT was positively associated with ASMI (*r* = 0.313, *p* < 0.001) and HGS (*r* = 0.264, *p* < 0.001) and negatively with TUG (*r* = −0.147, *p* < 0.001). Notably, a sex‐related difference was observed in the correlation between TMT and HGS, with a stronger association in males (male: *r* = 0.269, *p* < 0.001; female: *r* = 0.138, *p =* 0.003).

### 3.3 TMT Was Associated With Cognitive Decline

Spearman's partial correlation analysis, adjusted for age, sex and education, showed a positive correlation between TMT and general cognitive performance, as measured by MoCA‐B (*r* = 0.136, *p* < 0.001) and ACE‐III scores (*r* = 0.099, *p* = 0.006). MoCA‐B showed a stronger correlation with TMT than ACE‐III. Among cognitive domain tests, TMT was associated with the score of AVLT recognition (*r* = 0.078, *p* = 0.040), suggesting a potential role in core cognitive functions such as memory retrieval (Table S1). When adjusting TMT for height, height squared, weight or BMI, its associations with cognitive function were weakened (data not shown), suggesting that TMT may be influenced by these factors. Meanwhile, a similar analysis comparing TMT with ASMI revealed that ASMI had a significant but generally weaker association with cognitive function (Table S1). Given the stronger association of TMT with MoCA‐B, a more detailed analysis was carried out. Partial correlation analysis, adjusting for potential confounders including age, sex, education level, MNA‐SF score, history of hypertension and APOE genotype (Figure S2), confirmed the positive correlation between TMT and MoCA‐B (*r* = 0.124, *p* = 0.001; Table 2). Stratified analyses were performed based on sex, age, degree of cognitive impairment, APOE genotype and brain Aβ burden. Results stratified by sex and APOE genotype showed similar findings. Notably, stratification by age, cognitive status and brain Aβ burden revealed a significant association between TMT and MoCA‐B only in participants aged over 65 years, those with MCI and those without Aβ deposition (Table 2). In contrast, ASMI was not significantly related to MoCA‐B and showed only a weak correlation with ACE‐III (Table S2).

**TABLE 2:** Correlation between skeletal muscle mass indexes and MoCA‐B score.

	TMT		ASMI	
	*r* value	*p*	*r* value	*p*
Total	0.124	**0.001**	0.060	0.116
Sex				
Male	0.131	**0.035**	0.064	0.310
Female	0.129	**0.008**	0.067	0.164
Age				
< 65	0.080	0.165	0.095	0.101
≥ 65	0.164	**0.001**	0.040	0.434
Diagnosis				
NC	0.040	0.457	−0.010	0.851
MCI	0.221	**0.006**	0.003	0.963
ad	0.033	0.733	0.079	0.410
APOE genotype				
Non‐ε4 carrier	0.109	**0.015**	0.044	0.330
ε4 carrier	0.145	**0.048**	0.097	0.189
Aβ deposition				
Negative	0.148	**0.003**	0.078	0.122
Positive	0.110	0.071	−0.037	0.552

*Note:* Statistically significant comparisons are bolded. Spearman's partial correlation was used for correlation analysis in the total participants and different subgroups. The model corrects for six screened variables that influence the correlation: age, sex, education level, MNA‐SF score, history of hypertension and APOE genotype.

Abbreviations: Aβ, amyloid‐β; ad, Alzheimer's disease; APOE, apolipoprotein E; ASMI, appendicular skeletal muscle index; MCI, mild cognitive impairment; NC, normal cognition; TMT, temporal muscle thickness.

### 3.4 TMT Quartiles Were Associated With the Risk of MCI

To assess the association between TMT and ad continuum, binary logistic regression was conducted using TMT quartiles as an independent variable, and MCI and ad as dependent variables. As shown in Table 3, Model 1, which adjusted for age, sex and education level, showed that participants in the highest TMT quartile (Q4) had significantly lower OR for MCI (OR: 0.513, 95% CI: 0.310–0.850, *p* < 0.05) and ad (OR: 0.443, 95% CI: 0.236–0.832, *p* < 0.05) compared to those in the lowest quartile (Q1). However, the association persisted only for MCI (OR: 0.494, 95% CI: 0.287–0.849, *p* < 0.05) and not for ad after adjusting for additional variables like hypertension, nutritional risk and APOE genotype. Given the distinct associations observed between TMT and MoCA‐B in subgroups stratified by age and brain Aβ burden (Table 2), we further explored the relationship between TMT quartiles and MCI risk in these subgroups. The analysis showed that the significant association between TMT quartiles and MCI risk persisted across age groups, but was only significant in participants without brain Aβ deposition (Table 3).

**TABLE 3:** Associations between temporal muscle thickness quartiles and MCI/ad.

		Q1	Q2 vs. Q1	Q3 vs. Q1	Q4 vs. Q1
		Ref.	OR (95% CI)	OR (95% CI)	OR (95% CI)
Total	MCI				
	Model 1	1.00	**0.570 (0.352–0.922)** *	0.778 (0.488–1.238)	**0.513 (0.310–0.850)** *
	Model 2	1.00	0.630 (0.381–1.042)	0.831 (0.508–1.357)	**0.494 (0.287–0.849)** *
	ad				
	Model 1	1.00	0.849 (0.503–1.435)	0.669 (0.391–1.144)	**0.443 (0.236–0.832)** *
	Model 2	1.00	0.880 (0.488–1.587)	0.785 (0.434–1.418)	0.562 (0.281–1.122)
	MCI				
Age < 65	Model 1	1.00	0.514 (0.232–1.138)	0.631 (0.294–1.356)	**0.404 (0.118–0.869)** *
	Model 2	1.00	0.584 (0.253–1.350)	0.693 (0.306–1.569)	**0.430 (0.189–0.974)** *
Age ≥ 65	Model 1	1.00	**0.531 (0.289–0.977)** *	0.774 (0.431–1.388)	0.519 (0.264–1.019)
	Model 2	1.00	0.626 (0.331–1.183)	0.841 (0.453–1.562)	**0.395 (0.187–0.833)** *
	MCI				
Aβ‐PET (−)	Model 1	1.00	**0.521 (0.286–0.949)** *	0.584 (0.323–1.055)	**0.490 (0.259–0.925)** *
	Model 2	1.00	0.583 (0.309–1.098)	0.701 (0.373–1.317)	**0.466 (0.234–0.928)** *
Aβ‐PET (+)	Model 1	1.00	0.736 (0.305–1.778)	1.239 (0.534–2.877)	0.604 (0.244–1.497)
	Model 2	1.00	0.781 (0.313–1.944)	0.995 (0.415–2.385)	0.583 (0.223–1.524)

*Note:* Statistically significant comparisons are bolded. Model 1 corrects for three covariates: age, sex and years of education. Model 2 corrects for six screened variables that influence the correlation of TMT and MoCA‐B: age, sex, education level, MNA‐SF score, history of hypertension and APOE genotype.

Abbreviations: Aβ, amyloid‐β; ad, Alzheimer's disease; MCI, mild cognitive impairment; PET, positron emission tomography.

* *p* < 0.05 vs. Q1.

### 3.5 TMT Was Associated With Tau Pathology in the Brain

**FIGURE 2:**
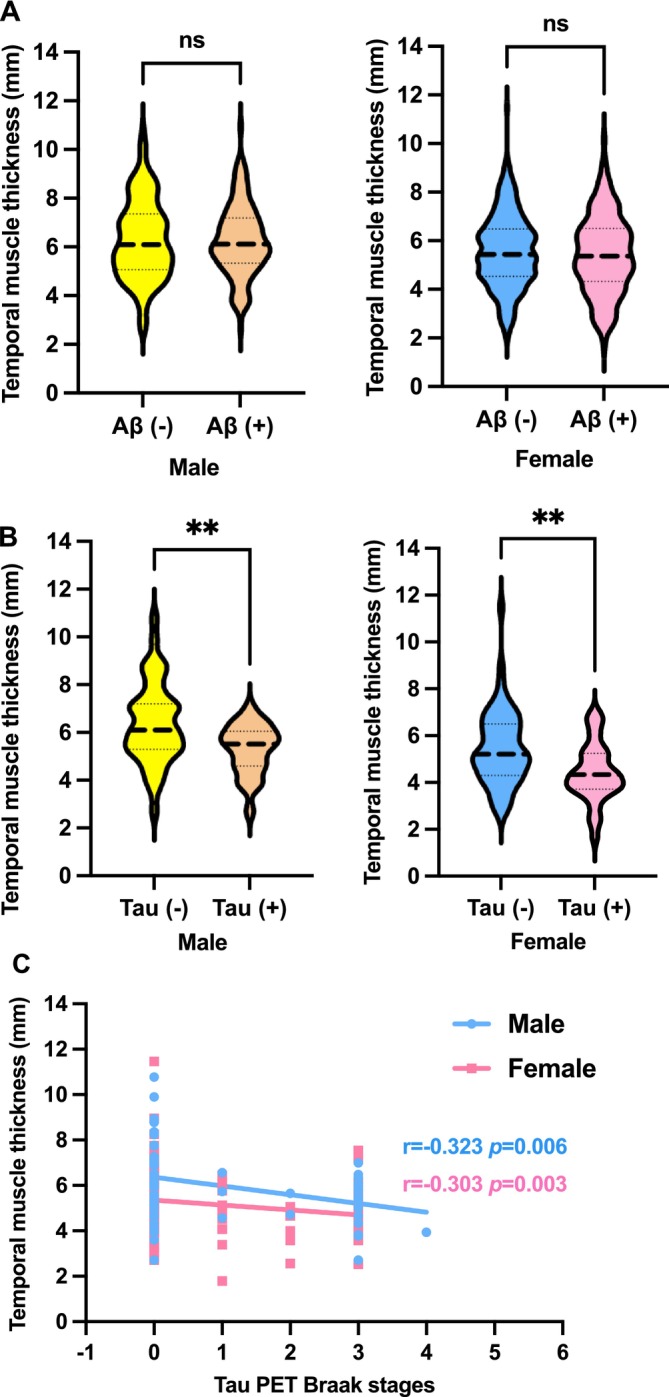
TMT is associated with brain tau deposition in both sexes. **p < 0.01.

To investigate whether TMT was associated with ad pathology, we examined the relationship between TMT and brain Aβ and tau deposition. Mean TMT was first compared between participants with and without brain Aβ deposition based on PET/CT imaging, and no difference was found in either sex (Figure 2A). We then randomly selected 165 Aβ‐PET‐positive participants for the measurement of brain tau deposition. TMT was significantly lower in those with positive tau deposition compared to those without (*p* < 0.01) (Figure 2B). Spearman's correlation analysis showed an inverse relationship between TMT and tau deposition, as assessed by tau Braak‐like stages (male: *r* = −0.323, *p* = 0.006; female: *r* = −0.303, *p* = 0.003) (Figure 2C). Additionally, TMT was negatively correlated with tau PET SUVR values in earlier Braak stages (Braak I: *r* = −0.232, *p* = 0.006; Braak II: *r* = −0.191, *p* = 0.026) after adjusting for age, sex and education (Table 4).

**TABLE 4:** Correlation between temporal muscle thickness and tau PET SUVR.

Tau PET Braak stage	*r* value	*p*
Braak I	−0.232	**0.006**
Braak II	−0.191	**0.026**
Braak III	−0.156	0.069
Braak IV	−0.125	0.145
Braak V	−0.125	0.145
Braak VI	−0.130	0.129

*Note:* Statistically significant comparisons are bolded. Partial correlation analyses were used by adjusting for age, sex and education.

Abbreviations: PET, positron emission tomography; SUVR, standard uptake value ratio.

### 3.6 TMT Was Associated With Functional Decline

Cognition is closely linked to functional performance. Thus, we investigated the relationship between TMT and functional outcome, as assessed by the FAQ. Stratified by FAQ score, TMT was significantly lower in the dependent group (FAQ score ≥ 5) compared to the independent group (FAQ score < 5) in both sexes (Figure S3). Multivariable logistic regression analyses, adjusted for age, sex and education, confirmed that lower TMT was associated with an increased risk of functional dependence (OR: 0.795, 95% CI: 0.684–0.922, *p* < 0.01). This association remained significant after further adjustment for HGS and TUG time (OR: 0.791, 95% CI: 0.670–0.935, *p* < 0.01) (Table 5).

**TABLE 5:** Binary logistic regression analyses for dependence in the study population.

	OR (95% CI)			
	Crude model	Model 1	Model 2	Model 3
TMT (mm)	**0.765 (0.669–0.875)** ***	**0.783 (0.676–0.906)** **	**0.795 (0.684–0.922)** **	**0.791 (0.670–0.935)** **
Age (year)		**1.039 (1.008–1.069)** *	**1.039 (1.007–1.071)** *	1.015 (0.979–1.052)
Sex (female)		0.705 (0.454–1.093)	**0.614 (0.391–0.962)** *	**0.336 (0.183–0.617)** ***
Education (year)			**0.883 (0.836–0.934)** ***	**0.914 (0.858–0.973)** **
HGS (kg)				**0.920 (0.885–0.957)** ***
TUG (s)				**1.216 (1.118–1.323)** ***

*Note:* Statistically significant comparisons are bolded. Model 1 corrects for age and sex, Model 2 further corrects for education and Model 3 further corrects for HGS and TUG.

Abbreviations: HGS, handgrip strength; TMT, temporal muscle thickness; TUG, Timed ‘Up and Go’ Test.

* *p* < 0.05.

** *p* < 0.01.

*** *p* < 0.001.

## 4 Discussion

In this study of a Chinese cohort aged 40–86, we explored the association between TMT and cognitive impairment, along with the underlying mechanisms. This study established three important findings. Firstly, we identified an independent link between thinner TMT and poorer cognitive performance, with thinner TMT serving as an independent risk factor for MCI, but not AD, suggesting its potential role in the preclinical stage of AD. In contrast, ASMI, a well‐established indicator of muscle mass, showed a weaker relevance to cognitive function. Secondly, we observed an association between TMT and cerebral tau deposition, suggesting that a decline in TMT may serve as a potential peripheral biomarker for cerebral tau deposition. Finally, thinner TMT was associated with impaired functional outcome, independent of muscle strength and physical function. Collectively, these findings suggest that TMT may be a crucial and early indicator of cognitive and functional decline.

Emerging evidence has hinted at a potential relationship between sarcopenia and cognitive dysfunction like MCI [27]; however, findings regarding the association between sarcopenia‐related traits, particularly muscle mass, and MCI or cognitive function remain inconsistent [28, 29]. Prior research on the connection between TMT and cognition is limited and exploratory, with two studies on AD patients yielding conflicting results regarding the association between TMT and the Mini‐Mental State Examination (MMSE) [5, 6], likely due to small sample sizes and insufficient control for confounders such as age, sex and education. In the present study, we analysed a larger sample (*n* = 147 for ad) with extensive adjustments for potential confounders, including age, sex, education level, nutritional risk, hypertension history and APOE genotype. Furthermore, we used MoCA‐B, a more sensitive tool than MMSE for detecting MCI, particularly in individuals with varying levels of cognitive function. Our findings demonstrated a close association between TMT and MoCA‐B, consistent with a recent study in Gothenburg (*n* = 657) showing a significant association between TMT and MMSE [3]. Different from previous studies, we comprehensively assessed cognitive function across both general cognition (MoCA‐B and ACE‐III) and multiple cognitive domains (memory, language, executive function and attention). TMT positively correlated with the scores of AVLT recognition, a useful marker for differentiating individuals with early cognitive decline from healthy controls and for predicting the progression of amnestic MCI in longitudinal studies [30]. Additionally, we investigated the potential link between TMT and the spectrum of AD, which includes MCI and AD, an area not previously examined. Consistent with the results in the subgroup analysis, we found that lower TMT was associated with a higher risk of MCI but not AD, suggesting that TMT may serve as an early biomarker for cognitive decline. Notably, the association between TMT quartiles and MCI was threshold‐dependent, not dose‐dependent, manifesting only when TMT declined below a certain critical cut‐off (e.g., Q4 in the present study). This finding was consistent with a 28‐year follow‐up study showing that BMI began to decrease 8 years before a dementia diagnosis [31], indicating low muscle mass in the preclinical phase of dementia. However, as sarcopenia progresses, it is likely that poor muscle function, rather than reduced muscle mass, may drive the association between sarcopenia and late‐life cognitive impairment [28], which could explain the negative association between TMT and AD, as well as the later tau PET Braak stage (III‐VI) SUVR values observed in our study. Interestingly, we did not observe any significant association between ASMI, a well‐established indicator of muscle mass, and MoCA‐B. This may be due to ASMI being estimated from a regression equation rather than being directly measured, which could be influenced by excess fat mass and potentially overestimate ASM in individuals with higher body weight, who are often associated with poorer cognitive status. This finding was in line with previous studies reporting no association between MMSE and ASMI measured by dual‐energy x‐ray absorptiometry (DXA) [3]. These inconsistencies across studies may be due to different muscle mass indicators [28, 32], highlighting that TMT could be a more reliable and distinct marker for identifying early cognitive decline and functional compromise in clinical settings.

In this study, we also conducted an exploratory analysis to investigate the potential mechanisms underlying the link between TMT and cognition, focusing on amyloid‐tau pathology in AD. Unlike previous studies that found associations between lower thigh muscle mass, BMI and cerebral Aβ retention in nondemented older adults [33, 34], we observed no such link between TMT and brain Aβ retention. However, our study provides the first epidemiological evidence of a significant connection between reduced muscle mass and abnormal tau deposition in adults with varying cognitive functions. Specifically, thinner TMT was associated with more severe tau Braak‐like stages in both sexes, and TMT was inversely related to tau PET SUVR values in the earlier Braak regions (transentorhinal, entorhinal and hippocampus) even after adjusting for age, sex and education. The aggregation of Aβ and tau in the pathophysiology of AD is a slow process, often detectable decades before clinical symptoms emerge [35]. While Aβ deposition is important, tau deposition is more predictive of future cognitive decline [12, 13] and functional impairment in the ad continuum [36]. This supports our findings on the relationship among TMT, tau deposition, cognitive impairment and functional performance. Our results suggest that the decline in TMT may correspond to a later stage of AD development, occurring after the Aβ‐positive but cognitively unimpaired phase. Incorporating TMT assessment into clinical practice could help identify individuals at high risk of cognitive deterioration, particularly those with MCI. The mechanism underlying the connection between muscle mass loss and cerebral tau burden remains unclear. One hypothesis is that misfolded tau protein accumulation in the motor system may contribute to muscle loss. In tauopathy and AD models, such as the tau P301S mice, the onset of muscle mass loss and mobility limitations suggests early signs of learning and memory deficits [37]. This suggests that tau deposition might have broader effects beyond the brain, potentially impacting skeletal muscle as well. However, evidence from hindlimb‐immobilised rats reveals a critical role of muscle mass loss in promoting tau deposition, insulin resistance and oxidative stress in the hippocampus. This relationship appears to be mediated, at least in part, by disruptions in systemic iron homeostasis, characterised by iron sequestration in disused muscle, accompanied by hypoferremia and decreased brain iron levels [38]. Given the cross‐sectional nature of our data, we cannot establish a causal relationship between TMT and tau deposition or fully elucidate the underlying mechanisms. Further investigation is warranted to better understand the impact of TMT on cognition and tau pathology.

Compared to commonly used methods like DXA and bioelectrical impedance analysis (BIA), TMT measurement offers several advantages: it is reliable, noninvasive, simple, fast and incurs no additional costs. Furthermore, in conditions that may influence muscle thickness, such as muscle oedema or atrophy, TMT measurement outperforms conventional methods. Unlike some indicators of muscle mass, TMT does not require adjustments for additional anthropometric parameters. Adjustments for height, weight or BMI can weaken the correlation between muscle mass and cognition, as observed in this study (data not shown). Overall, MRI‐based TMT measurement is a practical and cost‐efficient method for assessing skeletal muscle mass, cognition and functional performance in clinical settings.

To the best of our knowledge, this is the first study to explore the association between TMT, cognition and ad pathology in the brain. With a relatively large sample size, comprehensive adjustment for both established and potential confounders and inclusion of middle‐aged and older adults, this study presents pioneering and robust findings that can be generalised across age groups. However, there were several limitations. Firstly, the subset with tau PET imaging was relatively small, which limits statistical power and generalisability. Thus, our findings should be considered preliminary. Secondly, the cross‐sectional design of the study precludes any conclusions about temporality or causality. It remains unclear whether the decline in TMT precedes and contributes to tau deposition in the brain, or if the reverse is true. Longitudinal studies are therefore necessary to clarify the causal relationship between TMT and cognitive function. Thirdly, given that the C‐PAS study was not originally designed to address the objectives of this paper, we estimated muscle mass using an anthropometric equation rather than BIA or DXA, as recommended by the European Working Group on Sarcopenia in Older People (EWGSOP) and the Asian Working Group on Sarcopenia (AWGS). Nevertheless, this equation was previously validated in a Chinese population, achieving an adjusted *R* ^2^ of 0.90 and a standard error of estimate of 1.63 kg when compared to DXA [23].

## 5 Conclusion

In conclusion, this study underscored the possible link between MRI‐based TMT and cognition in middle‐aged and older adults, accompanied by an inverse relationship between TMT and tau pathology. TMT measurement may serve as a novel biomarker for identifying MCI due to AD and its associated functional impairments in clinical settings. However, further longitudinal research is needed to clarify the causal relationship between TMT and cognitive decline and to explore the mechanistic role of cerebral tau deposition in mediating the association between reduced TMT and cognitive impairments.

## Conflicts of Interest

The authors declare no conflicts of interest.

## Supporting information


**Figure S1.** Flowchart of the study population.
**Table S1.** Correlation between skeletal muscle mass indexes and a battery of standardised neuropsychological tests.
**Table S2.** Correlation between skeletal muscle mass indexes and ACE‐III score.
**Figure S2.** Confounders‐filtered forest map.
**Figure S3.** TMT is lower in the dependent group than in the independent group.
