# Supplementary material for: Temporal Muscle Thickness as a New Biomarker: Its Association With Alzheimer Pathology and Cognitive Impairment in Chinese Adults
Source: J Cachexia Sarcopenia Muscle. 2025 Jul 28;16(4):e70030. doi: 10.1002/jcsm.70030 (PMC12301632; doi:10.1002/jcsm.70030)
Supplement: Supplementary file 1 — Figure S1. Flowchart of the study population. Table S1. Correlation between skeletal muscle mass indexes and a battery of standardised neuropsychological tests. Table S2. Correlation between skeletal muscle mass indexes and ACE‐III score. Figure S2. Confounders‐filtered forest map. Figure S3. TMT is lower in the dependent group than in the independent group. [file JCSM-16-e70030-s001.docx]

***Journal of Cachexia, Sarcopenia and Muscle***

**Temporal muscle thickness as a new biomarker: its association with** **Alzheimer pathology and cognitive impairment in Chinese adults**

Yunxia Zhu^1^, Yingying Ke^1^, Chenxi Ren^1^, Jiehua Zhu^1^, Zhen Zhang^1^, Liang Cui^1^, Yuehua Li^2^, Fang Xie^3^, Qihao Guo^1^*

^1^ *Department of Gerontology, Shanghai Sixth People's Hospital Afﬁliated to Shanghai Jiao Tong University School of Medicine, Shanghai, China*

*PET Center, Huashan Hospital, Fudan University, Shanghai, China.*

^2^ *Department of Radiology, Shanghai Sixth People's Hospital Afﬁliated to Shanghai Jiao Tong University School of Medicine, No. 600, Yi Shan Road, Shanghai, China.*

^3^ *PET Center, Huashan Hospital, Fudan University, No. 518, Wu Zhong East Road, Shanghai 200233, China.*

****Corresponding to: Qi-hao Guo, Department of Gerontology, Sh**anghai Sixth People's Hospital* *Afﬁliated to Shanghai Jiao Tong University School of Medicine**,* *No. 600, Yi Shan Road, Shanghai, China.*

*E-mail: qhguo@sjtu.edu.cn (Q. Guo).*

**Fig. S1** Flowchart of the study population


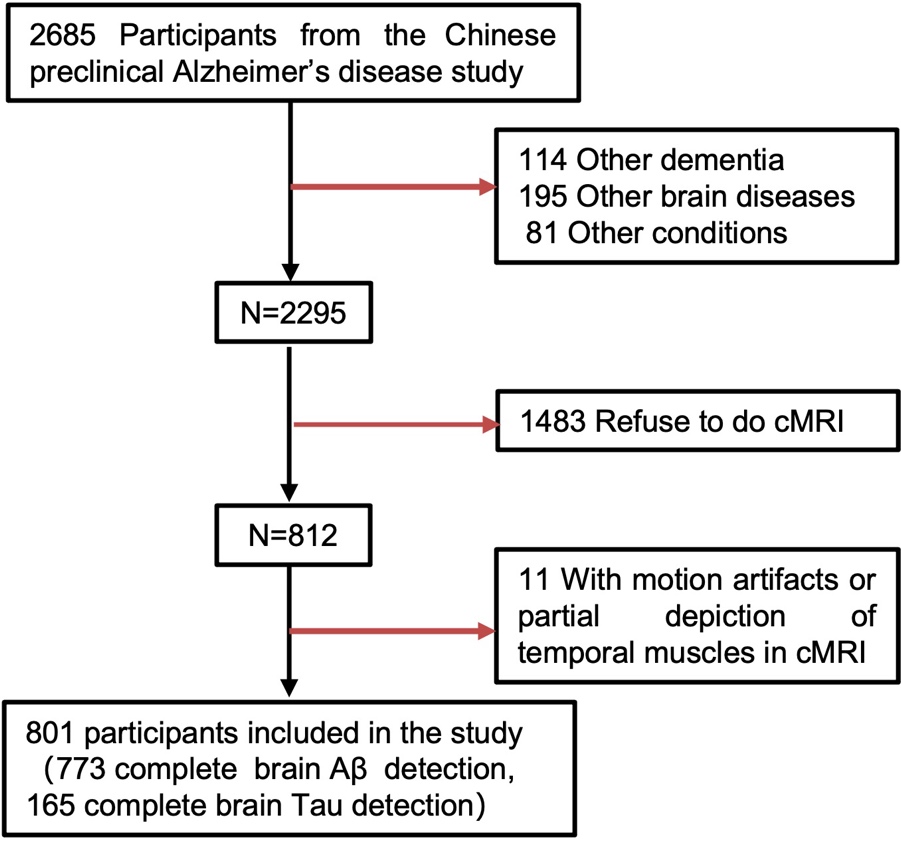


**Table S1** Correlation between skeletal muscle mass indexes and a battery of standardized neuropsychological tests

| **Variables** | TMT | |  | ASMI | |
| --- | --- | --- | --- | --- | --- |
|  | r value | *p* value |  | r value | *p* value |
| MoCA-B | 0.136 | **<0.001** |  | 0.101 | **0.006** |
| ACE-III | 0.099 | **0.006** |  | 0.118 | **0.002** |
| AVLT delayed recall | 0.016 | 0.637 |  | -0.002 | 0.965 |
| AVLT recognition | 0.078 | **0.040** |  | 0.033 | 0.395 |
| BNT | 0.048 | 0.206 |  | 0.019 | 0.626 |
| AFT | 0.052 | 0.156 |  | 0.056 | 0.141 |

Statistically signiﬁcant comparisons are bolded. Spearman partial correlation was used for correlation analysis, adjusting for age, sex, and years of education year. ACE-III, the Addenbrooke's Cognitive Examination; AFT, Animal Verbal Fluency Test; ASMI, appendicular skeletal muscle index; AVLT, Auditory Verbal Learning Test; BNT, Boston Naming Test; MoCA-B, Chinese version of Montreal Cognitive Assessment-Basic; TMT, temporal muscle thickness.

**Table S2** Correlation between skeletal muscle mass indexes and ACE-III score

|  | TMT | |  | ASMI | |
| --- | --- | --- | --- | --- | --- |
|  | r value | *p* value |  | r value | *p* value |
| Total | 0.061 | 0.109 |  | 0.082 | **0.031** |
| Sex |  |  |  |  |  |
| male | 0.119 | 0.058 |  | 0.049 | 0.435 |
| female | 0.031 | 0.525 |  | 0.105 | **0.030** |
| Age |  |  |  |  |  |
| <65 | 0.072 | 0.212 |  | 0.148 | **0.010** |
| ≥65 | 0.048 | 0.346 |  | 0.026 | 0.615 |
| Diagnosis |  |  |  |  |  |
| NC | 0.041 | 0.456 |  | 0.026 | 0.632 |
| MCI | 0.062 | 0.361 |  | -0.013 | 0.849 |
| AD | -0.118 | 0.198 |  | 0.169 | 0.065 |
| APOE genotype |  |  |  |  |  |
| Non-E4 carrier | 0.067 | 0.132 |  | 0.065 | 0.146 |
| E4 carrier | 0.053 | 0.472 |  | 0.120 | 0.103 |
| Aβ deposition |  |  |  |  |  |
| Negative | 0.074 | 0.140 |  | 0.071 | 0.161 |
| Positive | 0.069 | 0.259 |  | 0.037 | 0.547 |

Statistically signiﬁcant comparisons are bolded. Spearman partial correlation was used for correlation analysis in the total participants and different subgroups. The model corrects for six screened variables that influence the correlation: age, sex, education level, MNA-SF score, history of hypertension, and APOE genotype. Aβ, amyloid-β; AD, Alzheimer’s disease; APOE, apolipoprotein E; ASMI, skeletal appendicular muscle index; MCI, Mild Cognitive Impairment; NC, Normal Cognition; TMT, temporal muscle thickness.

**Figure S2** Confounders-filtered forest map


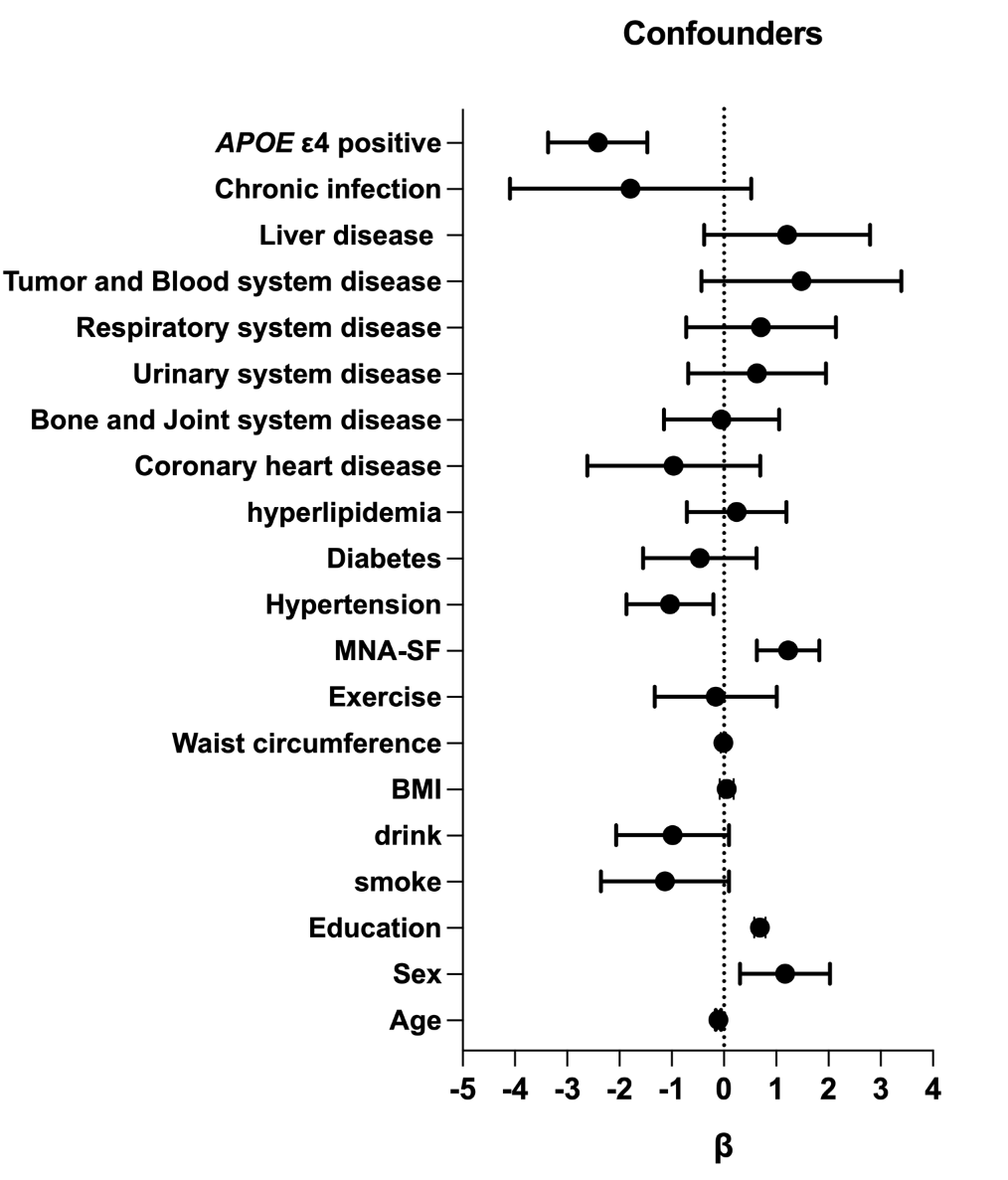


Note: The β coefficient and 95%CI were obtained by subtracting the coefficients of the two models from Bootstrap1000 times. Variables with a 95% confidence interval spanning over the value of 0 are identified as confounding factors affecting the relationship between temporal muscle thickness and cognition. APOE, apolipoprotein E; BMI, body mass index; MNA-SF, Mini-Nutritional Assessment Short-Form.

**Figure S3** TMT is lower in the dependent group than in the independent group


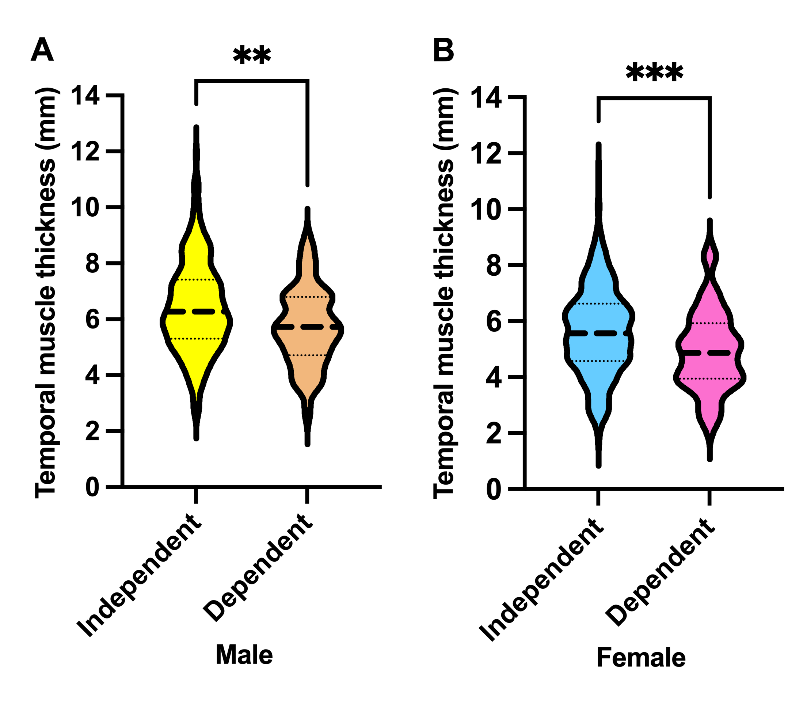


Note: TMT, temporal muscle thickness; the dependent group, FAQ score ≥5; the independent group, FAQ score <5.
